# Supplementary material for: Diversity, host-specificity and stability of sponge-associated fungal communities of co-occurring sponges
Source: PeerJ. 2018 Jun 4;6:e4965. doi: 10.7717/peerj.4965 (PMC5991299; doi:10.7717/peerj.4965)
Supplement: Supplemental Information 1 [file peerj-06-4965-s001.docx]

## Supplementary information

**Table S1:** Summary of isolation source and growth media of unique isolates (i.e. after identical isolate sequences were removed). Media: Peptone yeast glucose agar (PYG), Potato dextrose agar (PD) and Gause 1 agar (G1). Isolate sequence ID code (media_source_source-replicate-number_isolate-number). Isolate sequences are available through the NCBI GenBank, accession numbers are indicated in brackets.

| OTU_ID | *C. concentrica* (C) | *Scopalina sp.* (S) | *T. anhelans* (T) | Seawater (S) |
| --- | --- | --- | --- | --- |
| OTU_9 |  | PYG_S_2_05 (MG548542)  G1_S_2_03 (MG548546) |  |  |
| OTU_119 | PYG_C_3_02 (MG548512)  PYG_C_3_03 (MG548524)  PD_C_1_01 (MG548518)  PD_C_2_01 (MG548552) | PYG_S_2_03 (MG548534)  PYG_S_2_01 (MG548535)  PYG_S_3_04 (MG548533)  PYG_S_2_04 (MG548521)  PYG_S_1_01 (MG548529)  PD_S_2_02 (MG548537)  PYG_S_2_02 (MG548536)  G1_S_2_01 (MG548527)  G1_S_2_02 (MG548543)  PYG_S_3_02 (MG548541)  PD_S_1_01 (MG548523)  PYG_S_3_01 (MG548547)  G1_S_3_01 (MG548551)  PD_S_2_01 (MG548545) | PD_T_3_01 (MG548514)  G1_T_2_02 (MG548532)  G1_T_1_01 (MG548550)  PD_T_1_01 (MG548519) | PYG_SW_2_01 (MG548513)  G1_SW_3_01 (MG548544) |
| OTU_165 | G1_C_3_01 (MG548531)  PYG_C_2_02 (MG548516)  PGY_C_3_01 (MG548539)  PYG_C_2_01 (MG548540)  G1_C_1_01 (MG548553) | PD_S_2_03 (MG548517) |  |  |
| OTU_264 | PD_C_3_01 (MG548530)  PD_C_2_02 (MG548525)  PYG_C_2_03 (MG548522) | G1_S_1_01 (MG548538) |  | G1_SW_3_02 (MG548526) |
| OTU_265 |  | PYG_S_3_03 (MG548515) |  |  |
| OTU_266 |  |  | PYG_T_3_01 (MG548528)  G1_T_2_01 (MG548520) |  |
| OTU_268 |  |  | PYG_T_1_01 (MG548549) |  |
| OTU_269 |  |  | PYG_T_1_02 (MG548548) |  |

**Table S2:** Alpha diversity of whole fungal community as measured by the mean Shannon’s index ± standard deviation (SD). Comparison of diversity between samples were calculated with ANOVA and multiple comparison with Tukey’s test of subsampled variable/core fungal communities at an OTU-level clustered at 97%. Significant p-values (≤0.05) are shown in bold.

| Temporal samples | Shannon’s index (P-values) |
| --- | --- |
| 2014 vs. 2016 | 0.29 ± 0.44 vs. 1.43 ± 0.60 (**0.0005)** |
| *C. concentrica* 2014 vs. 2016 | 0.33 ± 0.49 vs. 1.54 ± 0.79 (0.15) |
| *Scopalina sp.* 2014 vs. 2016 | NA vs. 1.36 ± 0.037 (NA) |
| *T. anhelans* 2014 vs. 2016 | 0.49 ± 0.66 vs. 0.90 ± 0.27 (0.97) |
| Seawater 2014 vs. 2016 | 0.01 ± 0.018 vs. 1.89 ± 0.58 (**0.02)** |
| Sample type |  |
| Seawater vs. *Scopalina sp.* | 1.13 ± 1.11 vs. 1.36 ± 0.037 (0.99) |
| *T. anhelans* vs. *Scopalina sp.* | 0.77 ± 0.44 vs. 1.36 ± 0.037 (0.81) |
| *T. anhelans* vs. Seawater | 0.77 ± 0.44 vs. 1.13 ± 1.11 (0.87) |
| *Scopalina sp.* vs. *C. concentrica* | 1.36 ± 0.037 vs. 0.94 ± 0.89 (0.92) |
| Seawater vs. *C. concentrica* | 1.13 ± 1.11 vs. 0.94 ± 0.89 (0.98) |
| *T. anhelans* vs. *C. concentrica* | 0.77 ± 0.44 vs. 0.94 ± 0.89 (0.98) |

**Table S3:** PERMANOVA of the whole fungal community (sub-sampled to 250 reads) of sponges and seawater samples over time. Significant p-values (≤0.05) are highlighted in bold.

| Temporal samples | P-value base on relative abundance | P-value based on presence-absence |
| --- | --- | --- |
| 2014 vs. 2016 | **0.017** | **0.001** |
| *C. concentrica* 2014 vs. 2016 | 0.1 | **0.1** |
| *Scopalina sp.* 2014 vs. 2016 | NA | NA |
| *T. anhelans* 2014 vs. 2016 | 0.5 | 0.7 |
| Seawater 2014 vs. 2016 | 0.1 | **0.008** |
| Sample type |  |  |
| *C. concentrica* vs. *Scopalina sp.* | 0.4 | 0.51 |
| *C. concentrica* vs. *T. anhelans* | 0.29 | 0.24 |
| *C. concentrica* vs. seawater | 0.52 | 0.58 |
| *Scopalina sp*. vs. *T. anhelans* | 0.078 | **0.049** |
| *Scopalina sp.* vs. seawater | 0.39 | 0.12 |
| *T. anhelans* vs. seawater | 0.14 | 0.075 |

**Table S4:** Mean relative abundance percentages of OTUs in sponges (three species combined) and seawater with standard deviations (sd). Enrichment ratios are (OTU mean relative abundance in sponges/ OTU mean relative abundance in seawater). Sponge enriched OTUs are highlighted in bold.

| OTU_ID | Sponges (Sps)  mean % ± sd | Seawater (SW)  mean % ± sd |  |  | Enrichment ratio (Sps/SW) |
| --- | --- | --- | --- | --- | --- |
| OTU_3 | 9.09% ± 7.64 | 16.08% ± 25.96 |  |  | 0.6 |
| OTU_4 | 11.1% ± 8.23 | 8.34% ± 20.41 |  |  | 1.3 |
| OTU_5 | **8.2% ± 7.09** | **0.17% ± 0.19** |  |  | **48.2** |
| OTU_6 | **33.88% ± 10.19** | **17.28% ± 40.46** |  |  | **2.0** |
| OTU_7 | **3.01% ± 4.47** | **0.07% ± 0.09** |  |  | **40.5** |
| OTU_8 | **0.59% ± 1.83** | **0.04% ± 0.05** |  |  | **15.6** |
| OTU_10 | **6.21% ± 6.49** | **0.03% ± 0.03** |  |  | **246.9** |
| OTU_11 | **1.82% ± 2.9** | **0.07% ± 0.1** |  |  | **24.4** |
| OTU_12 | **1.8% ± 3.37** | **0.03% ± 0.05** |  |  | **51.6** |
| OTU_13 | **0.28% ± 1.09** | **0.05% ± 0.12** |  |  | **5.1** |
| OTU_14 | **1.77% ± 3.21** | **0.02% ± 0.04** |  |  | **78.7** |
| OTU_16 | 1.03% ± 2.45 | 2.98% ± 7.29 |  |  | 0.3 |
| OTU_18 | 1.37% ± 3.14 | 1.01% ± 2.43 |  |  | 1.4 |
| OTU_19 | 0.12% ± 0.76 | 1.25% ± 3.04 |  |  | 0.1 |
| OTU_21 | 0.17% ± 0.82 | 1.98% ± 3.07 |  |  | 0.1 |
| OTU_23 | 0.07% ± 0.6 | 3.33% ± 8.13 |  |  | 0.0 |
| OTU_24 | 0.08% ± 0.6 | 2.37% ± 5.81 |  |  | 0.0 |
| OTU_25 | 1.23% ± 2.87 | 3.87% ± 4.93 |  |  | 0.3 |
| OTU_27 | 0.14% ± 0.84 | 2.33% ± 5.71 |  |  | 0.1 |
| OTU_28 | 0.16% ± 0.76 | 4.13% ± 10.11 |  |  | 0.0 |
| OTU_37 | 0.06% ± 0.58 | 0.37% ± 0.89 |  |  | 0.2 |
| OTU_38 | 0.06% ± 0.6 | 1.17% ± 2.85 |  |  | 0.1 |
| OTU_40 | 0.09% ± 0.66 | 0.94% ± 2.31 |  |  | 0.1 |
| OTU_41 | 5.55% ± 6.38 | 0% ± 0 |  |  | - |
| OTU_45 | **0.21% ± 1.22** | **0.01% ± 0.01** |  |  | **24.5** |
| OTU_50 | 0.17% ± 1.04 | 1.01% ± 2.46 |  |  | 0.2 |
| OTU_51 | 0.05% ± 0.62 | 0.34% ± 0.56 |  |  | 0.2 |
| OTU_59 | 0.08% ± 0.67 | 0.09% ± 0.23 |  |  | 0.9 |
| OTU_60 | 0.09% ± 0.61 | 0.19% ± 0.46 |  |  | 0.5 |
| OTU_63 | **5.76% ± 6.39** | **0.01% ± 0.02** |  |  | **516.4** |
| OTU_87 | **0.05% ± 0.47** | **0.01% ± 0.01** |  |  | **7.6** |
| OTU_112 | 5.7% ± 6.37 | 0% ± 0 |  |  | - |
| OTU_142 | 0.03% ± 0.43 | 0.1% ± 0.25 |  |  | 0.3 |

**Table S5:** BLASTn results of the ‘variable/core’ fungal community ITS-amplicon representative sequences clustered at 97% similarity against the NCBI non-redundant database.

| OTU_ID | Taxon classification UPARSE program | Accession number | Best match | Isolation source | E-value | Query cover-age (%) | Max identity (%) |
| --- | --- | --- | --- | --- | --- | --- | --- |
| OTU_3 | (Genus) *Sporidiobolus sp.* | EF060745.1 | *Sporidiolales sp.* LM439 | Seawater | 1E-134 | 100 | 100 |
| OTU_4 | (Genus) *Epicoccum sp.* | MF972498.1 | *Epicoccum sp.* strain 1a | Archaeological marble | 1E-134 | 59 | 100 |
| OTU_5 | (Genus) *Aureobasidium sp.* | KY322539.1 | *Aureobasidium pullulans* | *Pinu heldreichii* fine roots | 1E-134 | 100 | 100 |
| OTU_6 | (Genus) *Cladosporium sp.* | KY781771.1 | *Cladosporium cf.* *cladosporoides* | Inland sea water | 1E-134 | 100 | 100 |
| OTU_7 | (Genus) *Curvularia sp.* | KY694447.1 | *Curvularia lunata* strain L7 | Cotton plant | 2E-113 | 100 | 96 |
| OTU_8 | (Genus) *Fusarium sp.* | EF488404.1 | *Fusarium sp.* XL-D19 | Annona squamosa L. | 5E-133 | 100 | 99 |
| OTU_10 | (Order) Leosporales | KF800567.1 | Uncultured fungus clone CMH476 | Indoor air | 1E-133 | 58 | 100 |
| OTU_11 | (Kingdom) Fungi | KP889871.1 | Uncultured fungus clone SG020_F01 | Soil | 1E-13 | 23 | 90 |
| OTU_12 | (Order) Agaricales | KC753423.1 | Unclutured *Calyptlla* clone R1_21 | Environmental samples | 6E-126 | 100 | 98 |
| OTU_13 | (Genus) *Beauveria sp.* | AY261369.1 | *Beauveria feline* strain CBS 250.34 | Isolate | 4E-90 | 100 | 89 |
| OTU_14 | (Class) Agaricomycetes | JQ007418.1 | Uncultured fungus clone | Environmental samples | 1E-134 | 100 | 100 |
| OTU_16 | (Genus) *Aspergillus sp.* | LN898703.1 | *Aspergillus jensenii* | Clinical samples | 1E-134 | 100 | 100 |
| OTU_18 | (Genus) *Mycospaerella sp.* | JQ732918.1 | *Mycosphearella sp.* AA-2012 | Leaves | 1E-134 | 100 | 100 |
| OTU_19 | (Kindom) Fungi | KU163812.1 | Uncultured fungus clone S12T_70 | Environmental sample | 5E-70 | 99 | 84 |
| OTU_21 | (Genus) *Mortierella sp.* | LC133885.1 | *Mortierella sp.* JCM 8527 | Soil | 3E-16 | 22 | 95 |
| OTU_23 | (Genus) *Metarhizium sp.* | MG230328.1 | *Metarhizium sp.* clone A17-S | (Plant) *Spiranthes novae-zelandiae* | 1E-134 | 100 | 100 |
| OTU_24 | (Kingdom) Fungi | JX384714.1 | Uncultred fungus clone 109A72941 | Environmental samples | 8E-42 | 100 | 75 |
| OTU_27 | (Genus) *Cryptococcus sp.* | HG937039.1 | Uncultured *Cryptococcus* | Soil | 3E-129 | 100 | 99 |
| OTU_28 | (Genus) *Cryptococcus sp.* | AY301025.1 | *Cryptococcus victoriae* strain S762 | *Vishniacozyma victoriae* | 1E-116 | 100 | 96 |
| OTU_37 | (Class) Lecanoromycetes | PK954735.1 | Uncultured *Glomus* clone cr-4 64201 | Soil | 7E-24 | 51 | 87 |
| OTU_38 | (Genus) *Coriolopsis sp.* | KJ093492.1 | *Coriolopsis trogii* isolate LG1 | Sapwood | 5E-133 | 100 | 99 |
| OTU_40 | (Class) Lecanoromycetes | JX042727.1 | Uncultured fungus clone G1_FF11 | Environmental samples | 7E-17 | 32 | 85 |
| OTU_41 | (Genus) *Alatospora sp.* | HM069492.1 | Uncultured fungus clone Fungi_Clone_89 | Pine forest soil | 1E-115 | 100 | 100 |
| OTU_45 | (Class) Lecanoromycetes | KM247690.1 | Uncultured fungus clone OTU51 | Environmental samples | 9E-16 | 22 | 95 |
| OTU_50 | (Class) Lecanoromyctes | MF966060.1 | Fungal sp. clone | Ponds | 6E-18 | 25 | 94 |
| OTU_51 | (Division) Ascomycota | MF965433.1 | Fungal sp. clone | Ponds | 3E-16 | 21 | 97 |
| OTU_59 | (Genus) *Malassezia sp.* | KM454161.1 | *Malassezia globose* strain 149.1 | *Homo sapiens* | 1E-134 | 100 | 100 |
| OTU_60 | (Kingdom) Fungi | KP889956.1 | Uncultured fungus clone SG071_D04 | Soil | 2E-18 | 23 | 97 |
| OTU_63 | (Class) Leotiomycetes | JN396554.1 | Uncultured fungus clone U_QM_090722_33_Aa01.b1 | Environmental samples | 1E-20 | 33 | 89 |
| OTU_87 | (Class) Lecanoromycetes | FN397125.1 | Uncultured fungus | Environmental samples | 7E-17 | 21 | 97 |
| OTU_112 | (Class) Agaricomycetes | MF965844.1 | Fungal sp. clone | Ponds | 3E-15 | 22 | 94 |
| OTU_142 | (Class) Agaricomycetes | MF965546.1 | Fungal sp. clone | Ponds | 3E-21 | 71 | 97 |
